# Supplementary material for: The Efficacy of Common Household Cleaning Agents for SARS-CoV-2 Infection Control
Source: Viruses. 2022 Mar 29;14(4):715. doi: 10.3390/v14040715 (PMC9026400; doi:10.3390/v14040715)
Supplement: Supplementary file 1 [file viruses-14-00715-s001.zip › viruses-1644865-supplementary.pdf]

viruses

MDPI

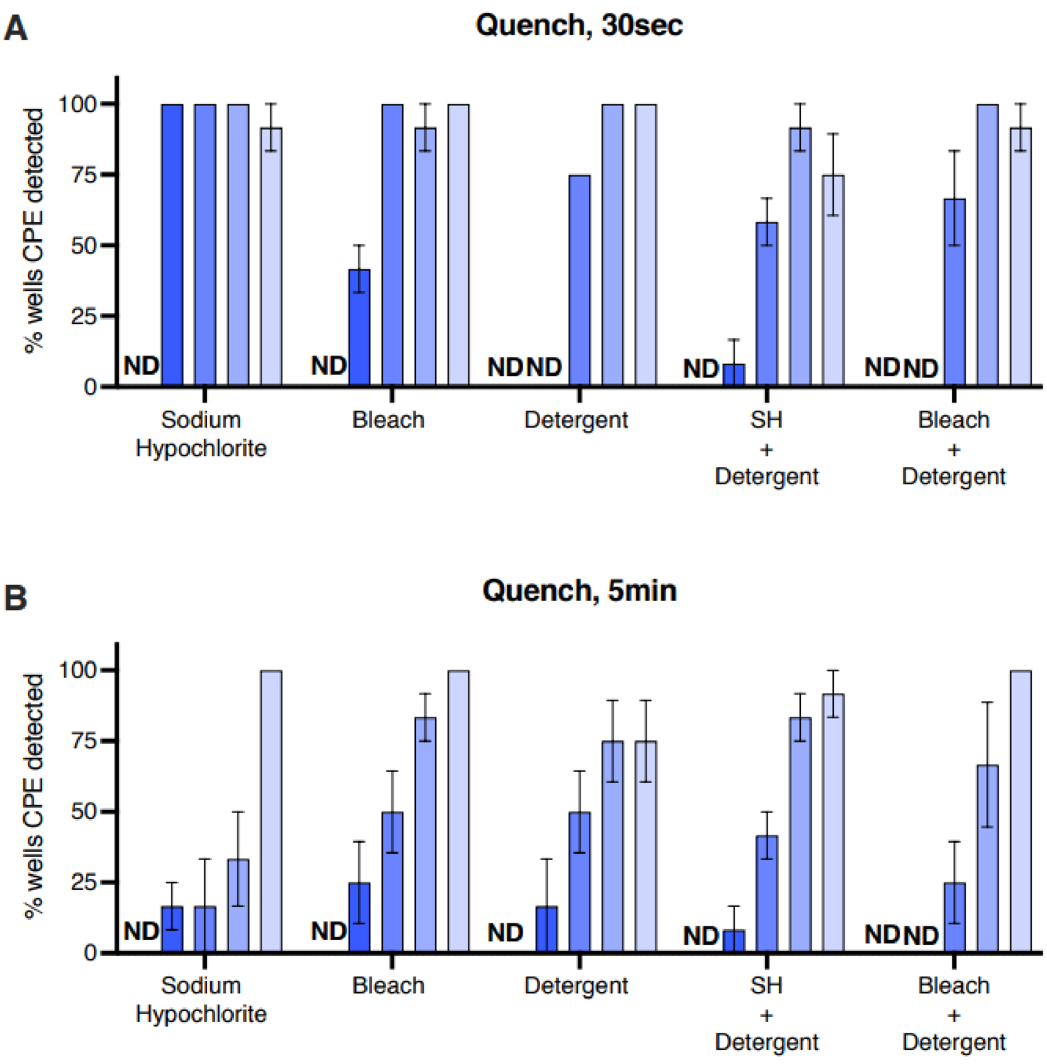

		Concentration	
	Dilution	Bleach/SH (g/L)	Detergent (%v/v)
■	1/4	10.5	2.5
■	1/80	0.525	0.125
■	1/800	0.0525	0.0125
■	1/8,000	0.00525	0.00125
□	1/80,000	0.000525	0.000125

Supplementary Figure S1. Preparation of bleach in infection media adversely affects the virucidal 718 concentration. Using the quench assay to test virucidal activity, detergent was added to bleach or 719 sodium hypochlorite (SH) diluted in media (MEM + 10μM HEPES, 2 mM glutamine and 720 antibiotics) then then exposed to SARS-CoV-2 for (**A**) 30 sec or (**B**) 5 min. Data is pooled from 4 721 independent experiments. Graphs show mean ± SEM of % wells in which viral-induced CPE could 722 be detected. Data pooled from 4 independent experiments. ND = virus induced cytopathic effect 723 was not detectable.
